# On investigating drivers’ attention allocation during partially-automated driving

**DOI:** 10.1186/s41235-024-00549-7

**Published:** 2024-04-10

**Authors:** Reem Jalal Eddine, Claudio Mulatti, Francesco N. Biondi

**Affiliations:** 1 Human Systems Lab, University of Windsor, Windsor, ON Canada; 2 Dipartimento di Psicologia e Scienze Cognitive, Universita’ di Trento, Rovereto, Italia; 3 Applied Cognition Lab, University of Utah, Salt Lake City, Utah USA

## Abstract

The use of partially-automated systems require drivers to supervise the system functioning and resume manual control whenever necessary. Yet literature on vehicle automation show that drivers may spend more time looking away from the road when the partially-automated system is operational. In this study we answer the question of whether this pattern is a manifestation of inattentional blindness or, more dangerously, it is also accompanied by a greater attentional processing of the driving scene. Participants drove a simulated vehicle in manual or partially-automated mode. Fixations were recorded by means of a head-mounted eye-tracker. A surprise two-alternative forced-choice recognition task was administered at the end of the data collection whereby participants were quizzed on the presence of roadside billboards that they encountered during the two drives. Data showed that participants were more likely to fixate and recognize billboards when the automated system was operational. Furthermore, whereas fixations toward billboards decreased toward the end of the automated drive, the performance in the recognition task did not suffer. Based on these findings, we hypothesize that the use of the partially-automated driving system may result in an increase in attention allocation toward peripheral objects in the road scene which is detrimental to the drivers’ ability to supervise the automated system and resume manual control of the vehicle.

## Introduction

The Society of Automotive Engineers defines six levels of automated driving systems from fully-manual (level 0) to fully-automated (level 5) (SAE, 2021). A level 2 or partially-automated system maintains control of the vehicle’s longitudinal (speed) and lateral (lane position) behavior and the human driver is responsible for actively monitoring its functioning and resuming manual control whenever necessary. The presence of partially-automated systems is rapidly increasing with the share of vehicles equipped with partially-automated systems being estimated to reach 60% of new vehicles sold in 2025 (Statista, 2022). The adoption of these systems comes with intended safety benefits. For example, driving with a partially-automated system that is capable of maintaining the vehicle safely within the lane and at a safe distance from the vehicle in front may help mitigate the safety risks of driving under high workload resulting from poor visibility or congested traffic. With the introduction of partially-automated systems into passenger vehicles, this is estimated to significantly reduce crashes and fatalities on the road (Gajera et al., 2022).

Despite these tangible safety benefits, early research conducted on partially-automated systems paints a somewhat different picture. Automating manual tasks leads to the role of the human transitioning from that of *system operator* to that of *system supervisor.* The reduction in the human’s responsibilities coupled with the requirement to monitor the functioning of the now-automated task leads to a gradual yet steady decrease in the driver’s ability to sustain attention toward the primary task at hand. This phenomenon–known as vigilance decrement–is a temporal decline in vigilance task performance (Grier et al., 2003) and although it has largely been investigated in the aviation literature (Molloy & Parasuraman, 1996; Warm et al., 2008) it has more recently been applied to the issue of driving automation. For example, Greenlee et al. (2022) had participants drive a simulated vehicle in either manual or partially-automated mode while completing a hazard detection task. The detection task required them to press a button whenever they detected an unsafely stopped vehicle occupying the lane of travel, and not respond whenever the vehicle was stopped safely. Results showed that, whereas no changes in task performance were observed over time in manual mode, driving in partially-automated mode resulted in more false alarms–i.e., responding when the vehicle was safely stopped, and a reduced ability to discriminate hazards from non-hazards. This pattern, which the authors interpreted as a vigilance decrement, was also replicated in the study by Biondi et al. (2023). Participants completed a detection response task while driving a vehicle in either manual or partially-automated mode. Consistent with the findings by Greenlee et al. (2022), a steeper temporal decline in detection task performance was observed during partially-automated driving. For similar studies, see Korber et al. (2015), McWilliams and Ward (2021) and Solis-Marcos et al. (2017).

Altogether, these findings suggest that when the vehicle automation is on, this may lead to drivers reducing their engagement in the task of driving. Thus, the question arises as to how this will affect driver attention allocation. Noble et al. (2021) compared driver glances between manual and partially-automated driving. Relative to the condition when drivers were in charge of manually operating the vehicle, the introduction of automation led to more glances being directed away from the forward roadway and more time spent looking away from the road. Morando et al. (2021) found consistent patterns. In their study participants drove a vehicle in either manual or partially-automated mode. In manual mode, 76% of the total driving time was spent looking at the road. When the automated system was on this declined to 64%. Gaspar and Carney (2019) found consistent results in their study, wherein driving with the partially-automated system on resulted in drivers spending more time looking away from the forward roadway.

With drivers spending less time looking at the road, it raises the issue of whether this is coupled with a broader visual scanning of the environment or, rather, an active, attentional engagement in scenes located away from the forward roadway. Mack (2003) used the term *inattentional blindness* to describe the phenomenon whereby individuals fail to notice clearly visible objects (also see Simons, 2000; Wolfe et al., 2022). In the seminal study by Neisser (1979), that Simons and Chabris (1999) later replicated with a gorilla, participants viewed a video of two teams passing a ball. Although participants were accurate in reporting the number of ball passes, because their attentional focus was on either of the two teams, the vast majority of them was blind to the presence of a woman strolling through the basketball court holding an umbrella. Applied cognition research has borrowed this phenomenon to explain real-world situations wherein the attentional engagement in a secondary task increases the likelihood of missing clearly visible information. For example, Strayer et al. (2003) investigated inattentional blindness in the context of driver multitasking. Participants were instructed to drive in a simulated driving scenario with or without carrying a conversation on a cell phone. Using a surprise recognition task, at the end of the experiment participants were quizzed about the presence of roadside billboards in the driving scene to measure differences in attention allocation between single and dual-task driving. Although the probability of fixating billboards was unaltered across the two conditions, a decrement in surprise recognition task performance was observed during cell phone driving. This pattern was accounted for by the authors as the direct result of secondary task engagement impairing the drivers’ ability to process clearly visible objects in the road scene. A similar paradigm was adopted by White and O’Hare (2022) to investigate the effect of multitasking on attention allocation during a simulated flight task. In this study, pilots completed two simulated flights with or without engaging in a concurrent cellphone conversation. Similarly to what was found in Strayer et al. (2003), the ability to recognize clearly-visible objects declined in the dual-task condition, a pattern that the authors accounted for in terms of inattentional blindness.

A competing hypothesis is that, instead of resulting in just a broader scanning of the road environment, operating vehicle automation will instead increase the processing of driving-unrelated, potentially distracting events in the driving scene. Recent research on the effect of vehicle automation on drivers’ workload posit that the reduction in drivers’ responsibilities from manual to partially-automated driving–whereby the state of the human driver transitions from system operator to system supervisor (Cummings et al., 2013)–will lower cognitive workload (Mishler & Chen, 2023; Solis-Marcos et al., 2017; Figalová et al., 2024). With a lowering of cognitive load, it is hypothesized that drivers may become more susceptible to potentially distracting events in the driving scene. This hypothesis would align with the experimental work by Minamoto et al. (2015). In their study, participants completed a selective attention task under increasing levels of cognitive workload while being instructed to ignore visual distractors. Conditions of lower cognitive load magnified the interference produced by the visual distractors. Sörqvist et al. (2016) had participants complete a primary selective attention task while being presented with distractors. Consistent with the work by Minamoto et al. (2015), conditions of higher cognitive load suppressed the processing of peripheral, task-irrelevant information. If validated, this hypothesis would offer an explanation for the patterns found in the work by Noble et al. (2021) and Morando et al. (2021) wherein the lowering driving demands of partially-automated driving resulted in a greater engagement in secondary tasks and less frequent on-road glances.

With this in mind, objective 1 aims to better understand the cognitive underpinnings of partially-automated driving, and how the use of vehicle automation may affect changes in drivers’ visual attention. In the current study we have participants drive a simulated vehicle in manual and partially-automated mode. Drivers’ visual scanning of the environment is measured by means of a head-mounted eye-tracker. Potential differences in workload between the two modes are also measured via recording subjective ratings in mental workload. As an indirect measure of attention allocation, we use a two-alternative forced choice recognition task. It requires participants to complete a driving task without knowing that their memory of the driving scene will later be tested. Borrowing from the work of Strayer et al. (2003), roadside billboards are used as probes for the recognition task because, although safety-relevant information is not displayed, they tend to attract drivers’ attention especially during monotonous highway driving (Edquist et al., 2011; Wallace, 2003; Young et al., 2009). Based on the assumption that attentional processing is necessary to form explicit memories (Ballesteros et al., 2006; Mulligan, 1998), we posit that, should the use of partial automation lead to a broader visual scanning of the environment, no differences in recognition task performance will be observed between manual and partially-automated driving. On the other hand, should the interference of potentially distracting objects increase when the automation is active, a better performance in the recognition task will be observed in partially-automated mode. Our second objective builds on the work by Greenlee et al. (2022) and Biondi et al. (2023). In their studies, a temporal decline in vigilance is observed when the partially-automated system was on. It follows that this may also be accompanied by changes in visual attention. To answer this question, we will split both the manual and partially-automated drives in two time periods and investigate changes in eye-tracking and recognition task performance over time.

## Methods

### Participants

Twenty-seven volunteers (18 women) were recruited from the University of Windsor’s student and research staff population. Their average age was 26 years old and the standard deviation was 3.62. Requirements to participate in the study included: having a valid driver’s license; having normal or corrected to normal vision and hearing; not having been the at-fault driver in any accident in the two years prior to the study; not having consumed an unusual amount of caffeine in the eight hours prior to the study; having slept for at least 7 h the night prior to the study; not having consumed marijuana or illicit drugs in the 24-h period prior to the study. This study was covered under the University of Windsor’s Research Ethics Board protocol #23–052.

### Study design

A within-subject study design was used for this research. Independent variables were: driving mode (2 levels: manual and partial automation), and time period (2 levels: period 1 and 2). Participants drove a driving simulator in either manual or partially-automated mode. The order of the two drives was fully counterbalanced across participants. Each drive was also segmented into two time periods to investigate changes in visual scanning and recognition task performance over time. In keeping with the paradigm adopted by Strayer et al. (2003), roadside billboards were used as experimental stimuli to measure drivers’ scanning behavior and memory of the driving scene (see Equipment section for more details). Dependent variables included: total number of fixations on billboards; total and average fixation duration on billboards; number of billboards that were fixated (i.e., number of billboards that received at least one fixation); number of billboards correctly recognized in the two-alternative forced choice recognition task; conditional probability of recognizing billboards that were fixated; subjective ratings of mental workload. More information is provided in the equipment and procedure sections.

### Apparatus

#### Driving simulator

**Fig. 1:**
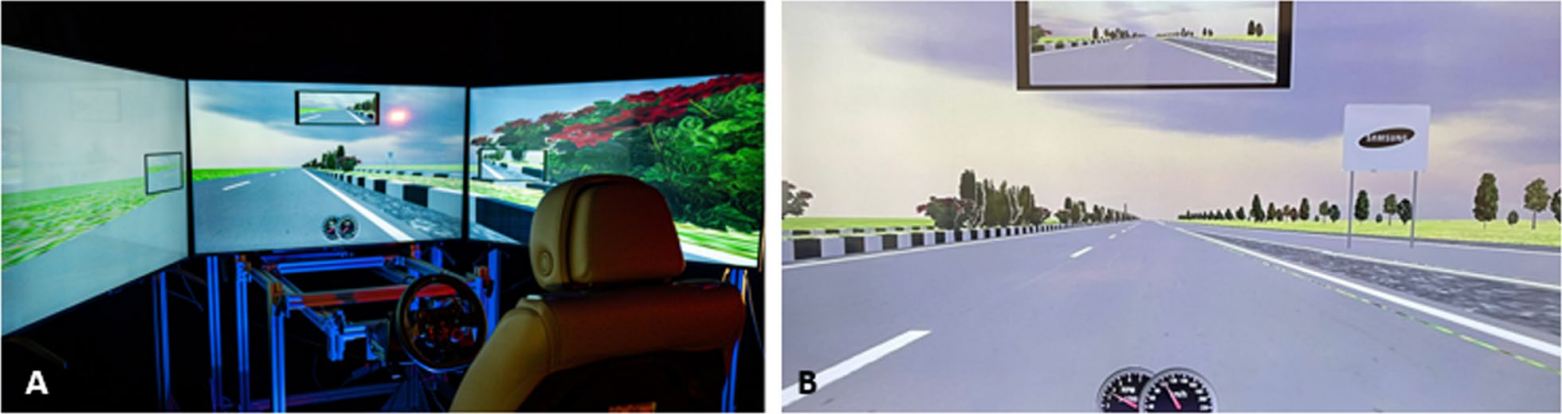
Photos of the driving simulator (**A**) and a driving scenario and billboard (**B**) used in the study

A medium-fidelity driving simulator running OpenDS 4.0 authoring and driving simulation suite was used for this study. The setup included: three 45-inch TV screens allowing for a 180-degree horizontal view of the driving environment; an adjustable leather seat from a Cadillac vehicle; Logitech G920 steering wheel and pedals (see Fig. 1). Images were rendered at 60 frames per seconds.

#### Driving scenarios

Participants drove on a straight, two-lane highway section with a length of 33.5 km that had a posted speed limit of 100 kph. Twenty-five oncoming vehicles with different characteristics (e.g., color, size) driving in the opposite lane were present in each 10-min scenario (manual and partial automation). No lead vehicle was present in either scenario. When driving in manual mode, participants were instructed to drive without the aid from any vehicle system at the posted speed limit of 100 kph. The average speed recorded in the study in manual mode was 100.45 kph (SD = 1.33 kph). When driving in partially-automated mode, in accordance with SAE (2021) the system maintained the vehicle at a speed of 100 kph and centered within the right lane. Participants were instructed to keep their hands on the wheel, remain attentive to the driving task, and resume manual control of the vehicle whenever necessary.

#### Billboard design

Ten billboards were located on the right side of the roadway at a distance of approximately 3 km from one another. Each billboard contained a picture of a popular brand’s logo in Canada. Brands selected for the study included those of popular car makers (e.g., Ford, Chrysler), food and beverage companies (e.g., Burger King, McDonald’s, Starbucks), and tech companies (e.g., Samsung). A total of twenty billboards each containing a unique logo (20 logos in total, one per billboard) were selected for this study, ten to be used during the manual drive and ten to be used during the partially-automated drive. Logos were in black and white, had a resolution of 1280 × 910 pixels, and had comparable sizes. Figure 1 shows an example of one of the billboards used in the study. Twenty billboards were used to create four different driving scenarios wherein the selection and the order of billboards was randomized. This was done to avoid presenting the same ten billboards during the manual drive and the same ten billboards during the partially-automated drive, and to reduce a related confounding effect. The order of the four scenarios was counterbalanced across participants. The decision to use roadside billboards was motivated by similar work by Strayer et al. (2003) and Sanbonmatsu et al. (2015). In Strayer et al. (2003)’s study, after driving a simulated vehicle in a low traffic density scenario, participants’ performance in a similar surprise recognition task was assessed to measure differences in drivers’ attention allocation toward driving scene objects between manual vs. cell phone driving. Sanbonmatsu et al. (2015) adopted similar stimuli to measure reductions in drivers’ self-awareness induced by conversing on a cell phone.

### Eye-tracker

A wearable, headmounted eye-tracker manufactured PupilLabs (Pupil Labs GmbH, Berlin, Germany) was used for this study. The eye-tracker uses three cameras: two eye cameras (one for each eye with a 120 Hz sampling rate), and one world camera recording from the participant’s perspective. The headset was connected to a desktop computer via a USB cable. A 9-point calibration was conducted prior to the study by having participants look at a 27-inch Lenovo monitor located approximately 80 cm away from the participant. Pupil Capture (v. 3.1.16) was used for the data recording, and Pupil Player (v 3.1.16) was used for data extraction.

### Recognition task

The PsychoPy software (version 2023.1.3) was used for the design and administration of the two-alternative forced choice recognition task. A total of 35 logos were randomly presented to participants during the recognition task: 20 logos that were used during the manual and partially-automated drives, and an extra 15, new lure logos that were not used in either drives. A logistic regression analysis was conducted to investigate a possible effect of order of presentation of the 35 logos on recognition task performance. The order of the logos was not found to have a significant effect on recognition task performance, χ^2^ = 0.405, p > 0.05. A preliminary test was conducted wherein a separate set of participants indicated no differences between the 35 brand logos (20 logos used for billboards in the two drives + 15 lure logos) in terms of their familiarity with them. All pictures of the logos were presented on a white background in the center of a 31.5-inch Acer screen. A black fixation cross presented in the center of the screen appeared for 2 s before each logo. Upon the presentation of each logo, participants were instructed to press the A key on the keyboard if they felt they encountered the picture during the drives, or the L key if they did not. Participants had unlimited time to make a selection.

### Subjective ratings of mental workload.

Subjective ratings of mental workload were measured by administering the NASA-TLX scale (Hart & Staveland, 1988). In its original form, the scale includes six subscales each measuring different facets of workload (mental demand, physical demand, temporal demand, effort, performance, frustration) on 21-point Likert scales from 1 (very low) to 21 (very high). Consistent with this study’s objective, only the ratings obtained for the mental demand scale are presented here.

## Procedure

The researcher met participants in the Human Systems Lab in the Human Kinetics building at the University of Windsor. First, participants were asked to fill out a screening questionnaire wherein they were instructed to provide information about their demographics, driving experience, vision and hearing, sleep habits, and the consumption of drugs and caffeine. Participants were then provided with an overview of the study. Note that information on the recognition task taking place at the end of the experiment was not provided at this time. Participants familiarized themselves with the driving simulator, and driving in manual and partially-automated mode for up to 15 min. The calibration process then took place where participants were instructed on what to do to calibrate the eye-tracker. It was conducted by having participants fixate on nine circles presented on a 27-inch Lenovo monitor located approximately 80 cm away from the participant. After the calibration was completed, participants were provided information on the experimental phase of the study. When driving manually, they were instructed to maintain the vehicle within the right lane and at a speed consistent with the posted speed limit of 100 kph. The driving scenario was designed so that participants would not encounter slow-moving vehicles in the lane of travel that required overtaking. When driving in partially-automated mode, participants were told that the automated system would control both the vehicle’s position and speed, and it was their responsibility to supervise the system functioning and resume manual control whenever necessary. Participants were instructed to keep their hands on the steering wheel and stay attentive toward the driving task. Participants took approximately 10 min to complete each drive. The order of drives (manual, partial automation) and scenarios (four different driving scenarios wherein the selection and the order of billboards was randomized) was counterbalanced across participants. At the end of the first drive, participants were instructed to complete the NASA-TLX scale whose completion took less than 1 min. Participants took a break of up to 5 min before the start of the second drive. At the end of the second drive, after the completion of the NASA-TLX scale, participants were instructed on how to complete the recognition task. They were told that they would be presented with a series of pictures that they may or may not have encountered in the experiment. They were instructed to press the A key if they encountered the picture or the L key if they did not. At the end of the recognition task, participants were debriefed and offered a $20 Amazon gift card for their participation in the study. The experiment took up to 1 h to complete.

### Data processing and analysis

#### Eye metrics

Pupil Lab Player was used for the extraction of gaze data. Five areas of interest (AOI) were defined for the eye metrics: rearview mirror, left and right sideview mirrors, forward roadway, and billboard. Given the objectives of this study, only the fixations directed to the billboard AOI were further analyzed. A fixation was defined as a cluster of gazes that were directed toward the same direction for a minimum duration of 150 ms. Such threshold for minimum fixation duration was set consistently with existing studies and to reduce the risk of misclassification (Blignaut, 2009; Camilli et al., 2008; Galley et al., 2015).Within the driving scenario, each billboard became visible to the driver 0.5 km away from the billboard’s location. Given the purpose of this study, only the data collected within this region was further analyzed. Note that no vehicles were present in the 0.5-km region before each billboard to avoid them acting as possible distractors. The total number of fixations on billboards was calculated as the total number of fixations directed at all billboards within each drive. Total and average fixation duration on billboards were calculated as the total time fixating on billboards and the average duration of each billboard fixation within each drive, respectively. The number of billboards that were fixated was calculated as the number of billboards within each drive that received at least one fixation. For objective 1, fixation metrics recorded in manual and partially-automated mode were compared. For objective 2, fixation metrics were split into two periods: 1 and 2. Period 1 included fixation metrics recorded for billboards 1 through 3 in each of the two drives. Period 2 included fixation metrics recorded for billboards 8 through 10 in each of the two drive. We decided to split the data this way to compare fixation metrics recorded at the beginning of the drive with those recorded at the end of the drive. Fixation data recorded for billboards 4 through 7 were not analyzed for objective 2.

#### Recognition task performance

Performance in the two-alternative forced choice recognition task was measured as follows. The total number of pictures that were correctly identified was calculated as the total number of pictures that were encountered in the drives and correctly identified as such (i.e., hits). In keeping with the work by Strayer et al. (2003), we also calculated the conditional probability of recognizing billboards that were fixated. This is an important metric in that it tests for memory of only the billboards that were scanned during the drive. Conditional probability was calculated as the percentage of billboards that were both fixated and recognized. Consistently with what was done for fixations, for objective 1 we compared recognition task performance recorded in manual and partially-automated mode. For objective 2, recognition task performance metrics were split into two periods: 1 and 2. Period 1 included performance metrics only for the billboards that were encountered at the beginning of each drive (billboards 1 through 3). Period 2 included performance metrics only for the billboards that were encountered at the end of each drive (billboards 8 through 10).

#### Data analysis

Linear models will be used for data analysis. Following the adoption of a full within-subject design, repeated-measure analyses of variance (ANOVA) will be used to investigate the effect of the two factors driving mode and time period on individual dependent measures. Paired t-tests will be conducted to investigate pairwise comparisons. α = 0.05 will be used as the probability threshold so that the null hypothesis is accepted or rejected whenever the probability associated with the statistical test is lower than or equal to α, or greater than α, respectively. Cohen’s *d* will be used as a measure of effect size for pairwise comparisons. For example, a *d* of 0.5 indicates that the two group means are 0.5 standard deviations apart. 95% confidence intervals will also be presented for pairwise comparisons. The processing and analysis of the data will be conducted using the *tidyverse*, *ezANOVA*, and *ggplot* libraries of Rstudio (version 2023.0.6).

## Results

Results are presented by objective. Objective 1 aims to investigate differences in drivers’ visual scanning and recognition task performance between manual and partially-automated driving. Objective 2 aims to investigate temporal changes in visual scanning and recognition task performance in the two modes.

### Differences in visual scanning and recognition task performance between manual and partially-automated mode (objective 1).

To explore differences in visual scanning between manual and partially-automated mode, separate paired t-tests are conducted with driving mode (2 levels) as the independent factor. The analysis conducted with the total number of fixations on billboards revealed a significant effect of mode, t(25) = 5.31, *p* < 0.001, 95CI [6.35, 14.41], Cohen’s d = 0.71, showing the number of fixations on billboards increased from manual driving (M = 12.30, SE = 2.24) to partially-automated driving (M = 22.62, SE = 2.39). This resulted in a longer total fixation duration in partially-automated mode (M = 6.26 s, SE = 0.93 s) relative to manual mode (M = 3.24 s, SE = 0.59 s), t(25) = 5.35, *p* < 0.001, 95CI [1.86, 4.19], Cohen’s d = 0.71. The total number of billboards that received at least one fixation also increased from manual (M = 4.15, SE = 0.52) to partially-automated mode (M = 5.80, SE = 0.54). Average fixation duration was also longer in partially-automated mode (M = 284.94 ms, SE = 20.46) relative to manual mode (M = 232.51 ms, SE = 21.22), t(25) = 2.11, *p* < 0.05, 95CI [1.28, 103.58], Cohen’s d = 0.49. Analysis also showed that the total time fixating driving-related areas (on-road, mirrors) was 65% in manual mode and 42% in partially-automated mode.

Recognition performance in the two-alternative forced choice recognition task was also analyzed. Preliminary analysis revealed that the recognition task performance recorded in both manual and partial automation differed from that expected from random guessing. Analysis conducted to compare differences in recognition performance between modes revealed that the total number of billboards being correctly recognized increased during partially-automated mode (M = 6.00, SE = 0.43) relative to manual driving (M = 4.43, SE = 0.42), t(25) = 3.26, *p* < 0.001, 95CI [0.57, 2.58], Cohen’s d = 0.73. Separate analyses revealed that participants recognized lure billboards as such (i.e., correct rejections) 91% of the times. Analyses conducted on conditional probability showed a greater conditional probability during partially-automated mode (M = 70.27%, SE = 5.97%) relative to manual mode (M = 51.47%, SE = 6.37%), t(25) = 2.49, *p* < 0.05, 95CI [3.72, 38.85], Cohen’s d = 0.67, indicating that, whereas the probability of recognizing billboards that were fixated was roughly 50% in the manual condition, driving with the automation on also led to drivers correctly recognizing 70% of the billboards that were scanned. Data are presented in Table 1.

**Table 1:** Mean (M) and standard error (SE) for fixation and recognition measures in manual and partially-automated mode

Measure		Mode	
		Manual	Partial automation
Total fixations at billboards	M	12.31	22.69
	SE	2.25	3.39
Total fixation duration (seconds)	M	3.24	6.27
	SE	0.58	0.93
Total number of billboards fixated	M	4.15	5.81
	SE	0.52	0.54
Average fixation duration (milliseconds)	M	232.51	284.94
	SE	21.22	20.46
Total number of billboards recognized	M	4.42	6.00
	SE	0.42	0.43
Conditional probability	M	51.48	72.77
	SE	6.38	5.98

Analyses conducted on subjective ratings of mental workload revealed significant differences between manual and partially-automated driving, t(25) = 6.19, *p* < 0.05, 95%CI[1.5, 3.0], Cohen’s d = 1.599. In particular, mean ratings declined from 19.33 (SD = 2.54) in manual mode to 10.26 (SD = 1.64) in partially-automated mode, a patter consistent with the work by Solis-Marco et al. (2017) and Biondi et al. (2023).

### Time changes in visual scanning and recognition task performance between modes (objective 2).

**Fig. 2:**
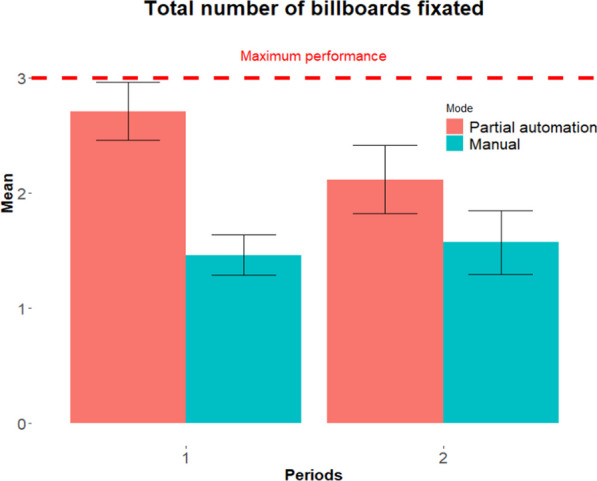
Total number of billboards fixated by mode and period. Three billboards fixated represent the maximum performance. Error bars represent standard error

Our second objective investigates possible time differences in visual scanning between modes. Analyses of variance (ANOVA) were conducted with driving mode (2 levels: manual, partial automation) and time period (2 levels: period 1 and 2) as within-subject factors and fixation and recognition metrics as dependent measures. Participants’ driving experience was added as a covariate to all analyses. Analyses conducted on fixation metrics revealed no significant effect of period for the total number of fixations on billboards, F(1, 25) = 1.66, *p* > 0.05, total fixation duration, F(1, 25) = 3.01, *p* > 0.05, or average fixation duration, F(1,25) = 3.63, *p* > 0.05. No significant period by mode interactions were found for these metrics. No significant effect of driving experience was found, F < 1. Analyses conducted on the total number of billboards fixated revealed a significant period by mode interaction, F(1, 25) = 4.27, *p* < 0.05. No significant effect of driving experience was found, F < 1. Post-hoc analyses revealed that, whereas no significant changes were found over time in manual mode, t < 0, a significant decline was found in partially-automated mode between period 1 (M = 2.71, SE = 0.25) and period 2 (2.11, SE = 0.29), t(25) = 2.35, *p* < 0.05, 95CI [0.13, 2.03], Cohen’s d = 0.37 (see Fig. 2).

**Fig. 3:**
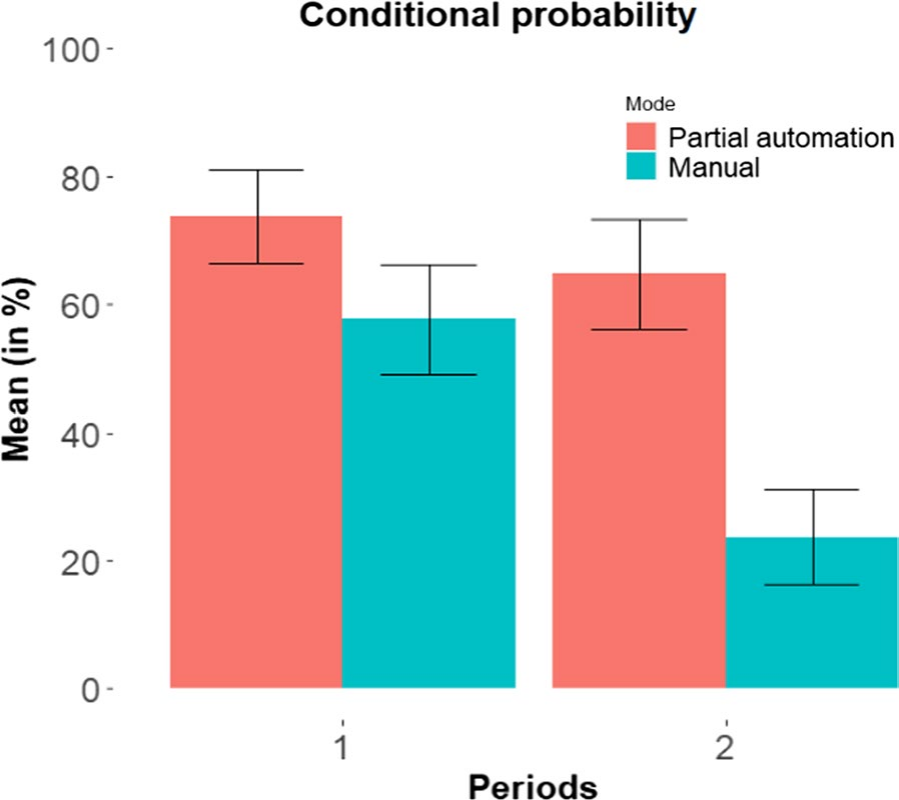
Conditional probability of recognizing billboards that were fixated by time period and mode. Error bars represent standard errors

Similar analyses were conducted to investigate recognition performance over time. Analyses conducted on the total number of billboards recognized revealed no significant effect of period nor period by mode interaction. Analyses conducted on the conditional probability of recognizing the billboards that were fixated showed a significant period effect, F(1,25) = 7.91, *p* < 0.001. No significant effect of driving experience was found, F < 1. Post-hoc analyses revealed that, whereas conditional probability remained steady over time in partially-automated mode, t < 1, it declined during manual driving, t(25) = 2.88, *p* < 0.001, 95CI [9.70, 58.24], Cohen’s d = 0.82 (see Fig. 3).

## Discussion

This study’s objective 1 was to investigate differences in drivers’ visual scanning and recognition task performance between manual and partially-automated mode. Results showed that, relative to manual driving, having automation on resulted in drivers spending more time looking away from the road at billboards. In partially-automated mode, we observed a greater number of fixations on billboards, longer total and average fixation duration, and more billboards receiving at least one fixation. With this in mind, the next step was to investigate drivers’ performance in the two-alternative forced choice recognition task. Relative to manual driving, the use of automation led to an increase in the billboards being correctly recognized. Whereas in manual mode drivers recognized an average of 4.43 billboards, this increased to 6 during partially-automated mode. This pattern was also confirmed by the analyses conducted on the conditional probability of recognizing those billboards that were fixated. Conditional probability also increased with automation, from roughly 50% in manual mode to 70% in partially-automated mode. A significant decline in subjective ratings of mental workload was also observed from manual to partially-automated driving.

These data are key to furthering our understanding of the safety implications of vehicle automation. The safe and effective operation of partially-automated systems requires the driver to stay vigilant while supervising the functioning of the automated system (SAE, 2021). Research by Noble et al. (2021), Morando et al. (2021), and Gaspar and Carney (2019) show that when the partially-automated is on, drivers tend to spend more time looking away from the road. Our data add to the literature on cognition in automation showing that this is not merely an issue of inattentional blindness whereby drivers spend more time gazing outside the forward roadway without processing information from the driving scene. Instead, the better performance in the recognition task observed in partially-automated mode indicates that drivers may also have been actively engaged in the processing of potentially distracting objects like roadside billboards. If this hypothesis holds true, the observed pattern is particularly perilous as it may further slow the switching of attention back to the primary task of driving, thus reduce the drivers’ ability to safely resume control of the vehicle.

These findings are also relevant for the literature in applied cognition. Analysis conducted on subjective workload show a reduction in mental workload when the automation was on, a finding that is consistent with prior work by McWilliams and Ward (2021) and Mishler and Chen (2023). Building on the experimental work by Minamoto et al. (2015) and Sorqvist et al. (2016) wherein the distraction potential of task-irrelevant visual stimuli was powered under lower load, it is then plausible that the decline in workload experienced during partially-automated driving may have increased the distraction potential of roadside billboards. This intepretation would also be consistent with the literature on cognitive tunnelling whereby a reduction in the drivers’ useful field of view is observed under greater cognitive load (Strayer et al., 2011; Vater et al., 2022). For example, Reimer (2009) had participants complete a driving task alongside a secondary cognitive task. As the cognitive load imposed by the secondary increased, a reduction in gazes directed toward the periphery was observed. Similar results were found by Biondi et al. (2015) wherein the occurrence of anticipatory glances aimed at inspecting the environment for potential peripheral hazards (e.g., pedestrians waiting at crosswalks) decreased under eleveated cognitive load. See Wolfe et al. (2019) for additional information on the combined effect of cognitive load and target location on peripheral vision.

This study’s second objective was to investigate time changes in visual scanning and recognition task performance between modes. Seminal research by Parasuraman and Warm (Grier et al., 2003; Warm et al., 2008) show that the ability to sustain attention over time decreases during vigilance tasks. Driving studies by Greenlee et al. (2022) and Biondi et al. (2023) also found that, relative to manual driving, having the partially-automated system engaged resulted in a steeper vigilance decrement. Building on this literature, we wanted to investigate how fixation and recognition performance changed over time in both manual and partially-automated mode. Analyses conducted on fixation metrics revealed a significant period by interaction mode for the total number of billboards fixated. In particular, whereas no changes were found over time during manual driving, a subtle yet significant decline was found in partially-automated mode. Analyses conducted on conditional probability offer more insight into the attentional underpinning of this trend. Whereas a decline in conditional probability was observed from period 1 to period 2 in manual mode, the probability of recognizing the billboards that were fixated remained steady over time in partially-automated mode. Combined with the fixation data, these results may suggest that, although drivers may have scanned fewer billboards toward the end of the drive, the attentional processing of these billboards did not suffer. The opposite trend was found for manual driving whereby, although no changes were observed in visual scanning over time, drivers seemed to be paying less attention to billboards toward the end of the drive, a pattern that might be interpreted as a vigilance decrement brought upon by monotonous driving (Larue et al., 2011; Schmidt et al., 2009).

Our study has limitations. First, participants were recruited from the University student and research staff population. They also did not drive a real vehicle but a simulated one and for only a limited amount of time. While we argue this does not undermine the validity of our findings–the use of driving simulators is common in applied cognition research (e.g.,Kaber et al., 2016; Kircher & Ahlstrom, 2012; Vaux et al., 2010), it is possible that, because simulated driving lacks the same level of realism and driver engagement than real-world driving, the time changes observed over the ten minutes of each drive might take longer to manifest when at the wheel of a real vehicle. It is also worth pointing out that, although a similar procedure was used by Strayer et al. (2003), the combination of fixation metrics and recognition task performance only serve as an indirect method for measuring attention allocation.

Our findings offer additional information on how using automation affects cognition. Despite the requirements of partial automation for drivers to stay attentive toward the task of driving, more evidence is showing the opposite to be true. As key previously manual tasks become automated, and the role of the driver transitions from operator to supervisor, this comes with clear consequences for safety. Building on seminal work by Parasuraman and Molloy (Molloy & Parasuraman, 1996; Parasuraman et al., 1993), Dunn et al. (2019) envisions three phases of vehicle automation use from novelty, wherein drivers learn about system capabilities, to experienced. According to this model, as drivers become more familiar with the functioning of the automated system and more comfortable relinquishing previously-manual operations to it, this comes with an increase in complacency manifesting in a gradual yet steady reduction in the driver’s ability to detect and adequately respond to system failures. Within the context of our findings, we argue that, with the transition from the novelty to the experienced phase, this leads to drivers paying even further attention to driving-unrelated objects in the surrounding environment.

## Data Availability

The datasets used and/or analysed during the current study are available from the corresponding author on reasonable request.
